# Importance of glomerular filtration rate change as surrogate endpoint for the future incidence of end-stage renal disease in general Japanese population: community-based cohort study

**DOI:** 10.1007/s10157-017-1463-0

**Published:** 2017-09-07

**Authors:** Eiichiro Kanda, Tomoko Usui, Naoki Kashihara, Chiho Iseki, Kunitoshi Iseki, Masaomi Nangaku

**Affiliations:** 1 Department of Nephrology, Tokyo Kyosai Hospital, 2-3-8 Nakameguro, Meguro, Tokyo, 153-8934 Japan; 2 Life Science and Bioethics Center, Tokyo Medical and Dental University, Tokyo, Japan; 3 Division of Health Service Promotion, The University of Tokyo, Tokyo, Japan; 4 Department of Nephrology and Hypertension, Kawasaki Medical School, Okayama, Japan; 5 Okinawa Heart and Renal Association, Clinical Research Support Center, Tomishiro Central Hospital, Okinawa, Japan; 6 Division of Nephrology and Endocrinology, Graduate School of Medicine, The University of Tokyo, Tokyo, Japan

**Keywords:** Surrogate endpoint, Glomerular filtration rate, End-stage renal disease, Dialysis, Chronic kidney disease

## Abstract

**Background:**

Because of the necessity for extended period and large costs until the event occurs, surrogate endpoints are indispensable for implementation of clinical studies to improve chronic kidney disease (CKD) patients’ prognosis.

**Methods:**

Subjects with serum creatinine level for a baseline period over 1–3 years were enrolled (*n* = 69,238) in this community-based prospective cohort study in Okinawa, Japan, and followed up for 15 years. The endpoint was end-stage renal disease (ESRD). The percent of estimated glomerular filtration rate (%eGFR) change was calculated on the basis of the baseline period.

**Results:**

Subjects had a mean ± SD age, 55.59 ± 14.69 years; eGFR, 80.15 ± 21.15 ml/min/1.73 m^2^. Among the subjects recruited, 15.81% had a low eGFR (<60 ml/min/1.73 m^2^) and 36.1/100,000 person years developed ESRD. Cox proportional hazards models adjusted for baseline characteristics showed that the risk of ESRD tended to be high with high rates of decrease in %eGFR changes over 2 or 3 years in the high- and low-eGFR groups. The specificities and positive predictive values for ESRD based on a cutoff value of %eGFR change of less than −30% over 2 or 3 years were high in the high- and low-eGFR groups.

**Conclusions:**

%eGFR change tends to be associated with the risk of ESRD. %eGFR change of less than −30% over 2 or 3 years can be a candidate surrogate endpoint for ESRD in the general Japanese population.

## Introduction

Chronic kidney disease (CKD) patients have high risks of death, cardiovascular disease (CVD), and end-stage renal disease (ESRD), which increase with the progression of CKD. Various endpoints have been used for clinical studies. The occurrences of death, CVD, and ESRD are important clinical endpoints for CKD patients, which are true endpoints, and are often used as the endpoints in clinical studies. However, because the events associated with these true endpoints are of limited frequency, the sample sizes based on the endpoints are large to evaluate the effects of a therapy on the endpoints, the study period are long, and a large budget is required. Thus, surrogate endpoints are used instead of these true endpoints for evaluating the effects of a therapy. The Biomarkers Definitions Working Group defined a surrogate endpoint as a parameter expected to predict the clinical benefits of a therapy on the basis of epidemiologic, therapeutic, pathophysiologic, or other scientific evidence [1]. The use of appropriate surrogate endpoint, which reflects kidney function and can quantify the risk of the true endpoints, may make the sample size of clinical studies smaller and the study period shorter.

The US National Kidney Foundation and the US Food and Drug Administration reported eGFR change as surrogate endpoint of ESRD [2–5]. %eGFR changes of less than −30% over 2 years have been proposed as a surrogate endpoint of ESRD and death [2, 6–8]. Although the applicability of %eGFR change as a surrogate endpoint has been examined in CKD patients in previous studies, it has not been evaluated yet in a general population. For this purpose, we examined whether %eGFR change can be used as a surrogate endpoint of ESRD using data of a community-based prospective cohort study in Okinawa, Japan. Thus, we (1) investigated the association between %eGFR change and the risk of ESRD, (2) determined the cutoff value that represents ESRD, and (3) evaluated the accuracy of %eGFR change for the prediction of ESRD.

## Materials and methods

### Data source

Medical records of participants who were screened from 1993 to 1996 by the Okinawa General Health Maintenance Association (OGHMA, currently Okinawa Health Promotion Foundation) were analyzed in this study [9–11]. The study population consisted of 69,727 subjects whose 1993 data were available. Subjects with missing data and outliers, and those who were already on dialysis were excluded from this study. Finally, 69,238 subjects whose 1993 data included eGFR were recruited in this study. Combined data from 1993 to 1996 were treated as baseline data. The outcome of this study was ESRD. Patients who started dialysis from the health examination within 15 years were identified from the Okinawa Dialysis Study registry, which covered the entire Okinawa geographic area [10]. No data of their death were found.

Baseline patient data including gender, age, systolic blood pressure, body mass index (BMI), serum creatinine and total cholesterol levels, and urinary protein positivity (dipstick test) were collected from all the subjects. Serum creatinine level was measured using a modified Jaffe reaction and converted to that determined by the enzymatic method [12]. eGFR was calculated using the equation for the Japanese population developed by Japanese Society of Nephrology (JSN) [13]. %eGFR changes over a baseline period (%/baseline years) were calculated based on the basis of two eGFRs using two-point datasets as follows:

$$\% {\text{eGFR changes over baseline years }} = \frac{{({\text{last eGFR }} - {\text{eGFR in }}1993) }}{{{\text{eGFR in }}1993}}\, \times \,100$$

To evaluate the difference in the calculation methods of %eGFR change, %eGFR change slope (%/year) was calculated by the least squares method using the combined datasets including serum creatinine levels at several points as follows:

$$\% {\text{eGFR change slope }} = \frac{\text{eGFR slope }}{{{\text{eGFR in }}1993}}\, \times \,100$$

### Statistical analyses

Statistical analyses were carried out separately on the groups classified on the basis of eGFR during the 1993 screening: the high-eGFR group, eGFR 60 or more ml/min/1.73 m^2^; the low-eGFR group, eGFR less than 60 ml/min/1.73 m^2^. The subjects were categorized on the basis of %eGFR changes by 10% from less than −40%. Variables are presented as mean ± standard deviation (SD). Urinary protein positivity was treated as a categorical variable. Intergroup comparisons of parameters were performed using the Chi-square test, *t* test or Mann–Whitney *U* test as appropriate. To evaluate the relationship between %eGFR changes and the risk of ESRD, the proportional hazard assumption in Cox proportional hazards models was confirmed using log[−log(survival)] versus log of survival time graphs and Schoenfeld residuals; after which, Cox proportional hazards models for time-to-event outcomes were examined. The multivariate Cox proportional hazards models with restricted cubic spline functions of %eGFR changes were adjusted for the baseline characteristics, namely, gender, age, BMI, systolic blood pressure, serum total cholesterol level, eGFR, and urinary protein level. Knots of the splines were placed at −30, −20, −10, 0 and 10% eGFR changes. The results are shown as hazard ratios (HRs) and 95% confidence intervals (CIs). Using 1000 datasets derived from the original dataset by the bootstrap method, we evaluated sensitivity and specificity with their 95% CIs for each cutoff value of %eGFR change. By taking the sensitivity, specificity, and prevalence of exposed subjects into consideration, we calculated positive predictive value (PPV). PPV was simulated on the basis of sensitivity and specificity with change in the prevalence of exposed subjects. PPV was calculated as follows:

$${\text{PPV }}(\% ) = \frac{{{\text{sensitivity}}\, \times \,{\text{prevalence}}\, \times \,100}}{{{\text{sensitivity}}\, \times \,{\text{prevalence}}\, + \, (1 - {\text{prevalence)}}\, \times \,(1 - {\text{specificity}})}},$$

where sensitivity and specificity were treated as fixed values for each cutoff value of %eGFR change. Statistical significance was defined as *p* < 0.05. These analyses were conducted using SAS version 9.4 (SAS, Inc., Cary, North Carolina), and R version 3.3.2 (The R Project for Statistical Computing, Vienna, Austria).

## Results

### Baseline characteristics

**Fig. 1:**
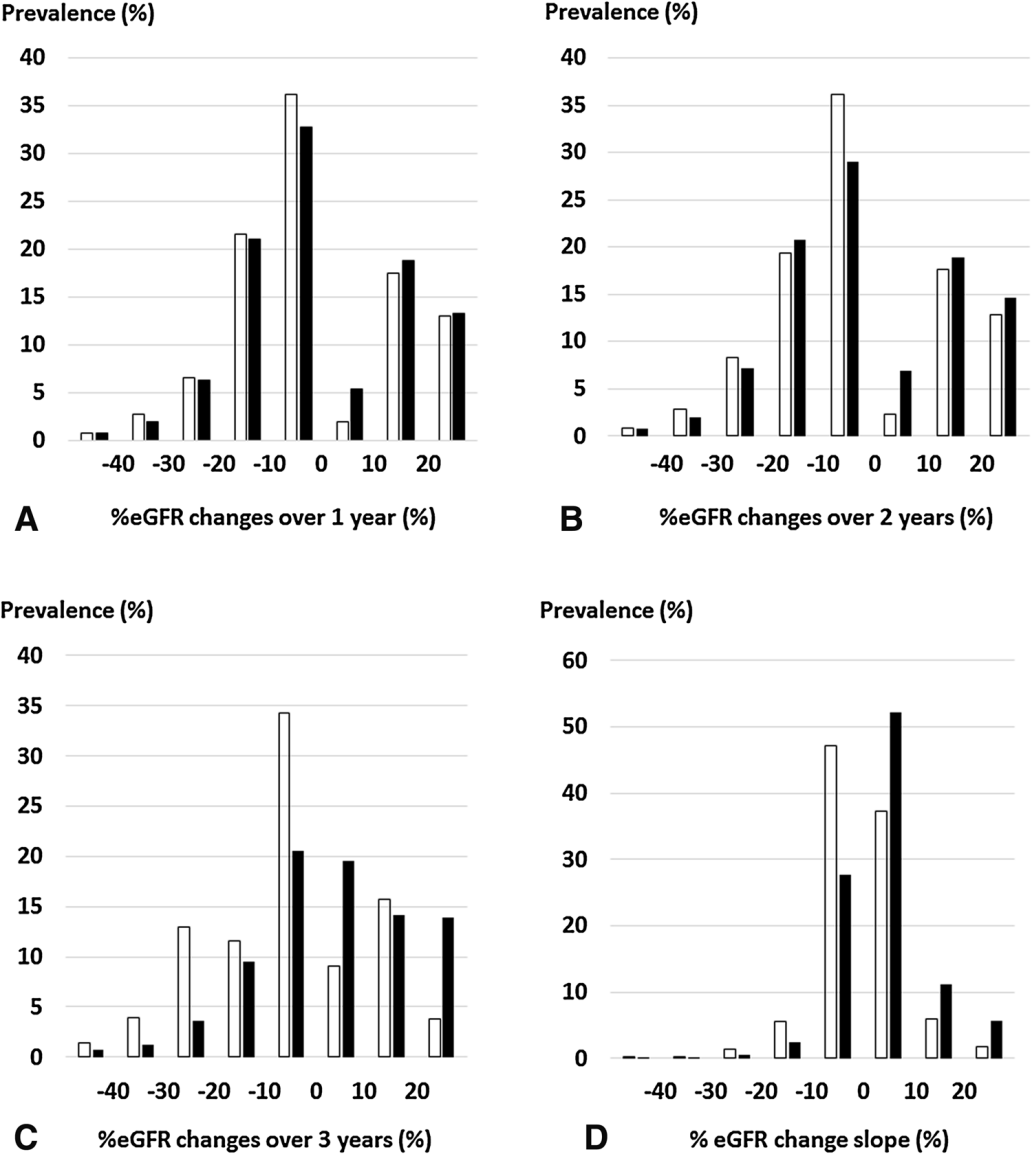
Distribution of %eGFR changes. **a** Distribution of %eGFR changes over 1 year, **b** distribution of %eGFR changes over 2 years, **c** distribution of %eGFR changes over 3 years, **d** distribution of %eGFR change slope. *White and black bars* show the distributions of %eGFR changes in the high- and low-eGFR groups, respectively. *eGFR* estimated glomerular filtration rate

The study population consisted of 69,238 subjects, 55,147 of whom had their 1994 data, 52,930 their 1995 data, and 49,848 their 1996 data. ESRDs were more frequently observed in the low-eGFR group than in the high-eGFR group (Table 1). The distributions of %eGFR changes are shown in Fig. 1. The mean of %eGFR changes in the low-eGFR group was higher than that in the high-eGFR group (Table 1; Fig. 1). In the high-eGFR group, the subjects with %eGFR changes of less than −40% over 1–3 years and %eGFR change slope were 0.67, 0.78, 1.43, and 0.11%, respectively. In the low-eGFR group, the subjects with %eGFR changes of less than −40% over 1–3 years and %eGFR change slope were 0.78, 0.74, 0.71, and 0.12%, respectively.

**Table 1:** Baseline and demographic characteristics of datasets

	All	High-eGFR group	Low-eGFR group	*p*
*n* (%)	69,238	58,292 (84.19)	10,946 (15.81)	
Male (%)	29,744 (42.96)	25,958 (44.53)	3786 (34.59)	0.0001
Age (years)	55.59 ± 14.69	53.19 ± 14.16	68.39 ± 10.15	0.0001
BMI (kg/m^2^)	24.08 ± 3.38	24.05 ± 3.4	24.27 ± 3.27	0.0001
SBP (mmHg)	127.8 ± 17.4	126.68 ± 17.17	133.77 ± 17.39	0.0001
Total cholesterol level (mg/dl)	204.3 ± 35.62	203.1 ± 35.45	210.68 ± 35.86	0.0001
Urinary protein				0.0001
– (%)	66737 (96.39)	56543 (97)	10194 (93.13)	
± (%)	1671 (2.41)	1224 (2.1)	447 (4.08)	
1+ or more (%)	830 (1.2)	525 (0.9)	305 (2.79)	
eGFR (ml/min/1.73 m^2^)	80.15 ± 21.15	85.37 ± 18.72	52.39 ± 6.97	0.0001
%eGFR changes over 1 year (%/year)	0.97 ± 19.38	0.78 ± 19.45	1.95 ± 18.97	0.0001
%eGFR changes over 2 years (%/2 years)	0.51 ± 20.87	0.12 ± 19.49	2.61 ± 27.04	0.0001
%eGFR changes over 3 years (%/3 years)	−0.34 ± 21.32	−2.39 ± 20.23	10.72 ± 23.52	0.0001
%eGFR change slope (%/year)	0.08 ± 9.18	−0.64 ± 8.70	3.91 ± 10.59	0.0001
ESRD (/100,000 person years)	383 (36.1)	186 (20.8)	197 (118.1)	0.0001

Values are expressed as mean ± standard deviation. The values were compared between the groups by the Chi-square test, *t* test or Mann–Whitney *U* test as appropriate

*BMI* body mass index, *SBP* systolic blood pressure, *eGFR* estimated glomerular filtration rate, *ESRD* end-stage renal disease

### %eGFR change and risk of ESRD

**Fig. 2:**
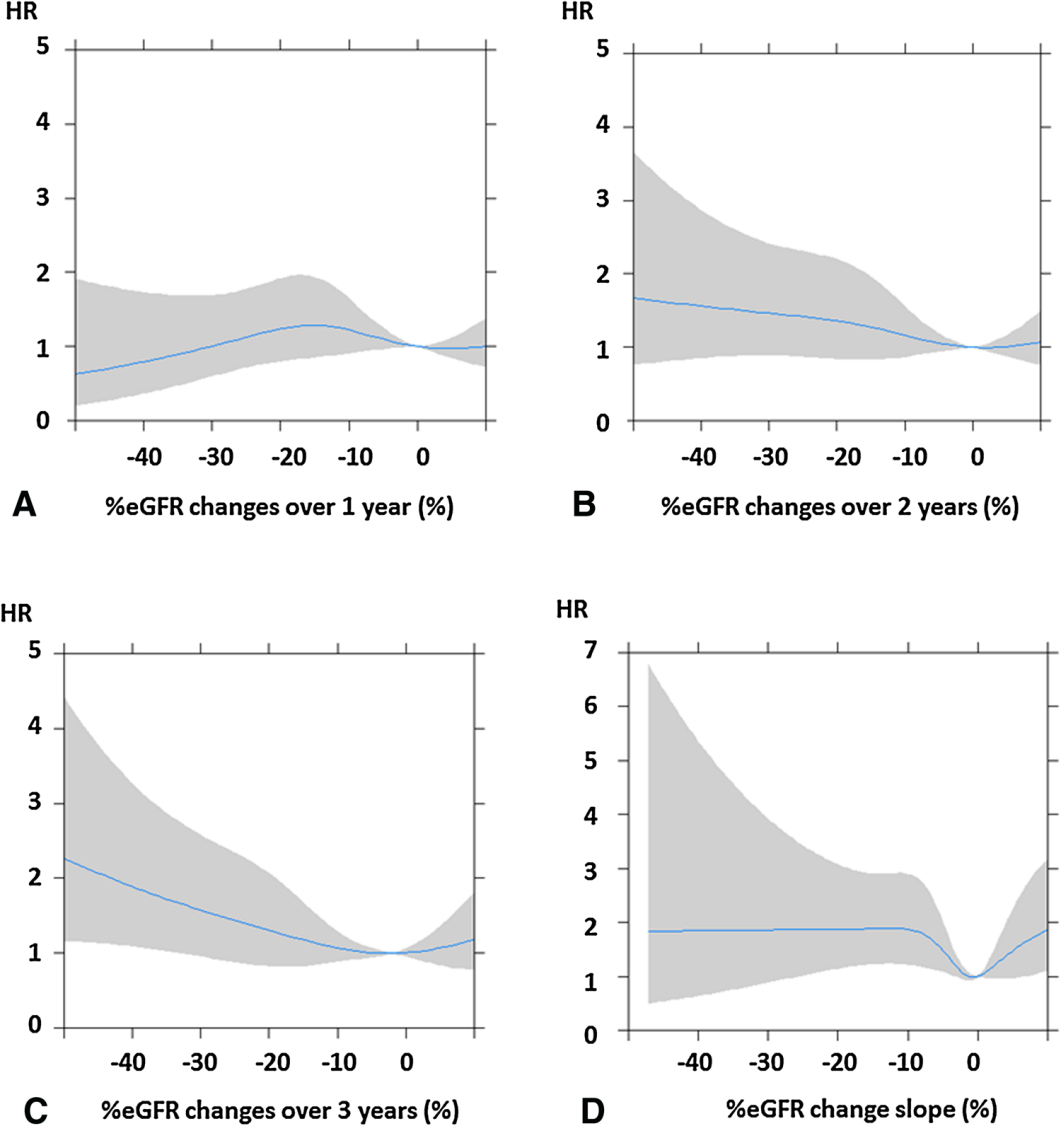
Relationships between %eGFR changes and risk of ESRD in the high-eGFR group. **a** %eGFR changes over 1 year, **b** %eGFR changes over 2 years, **c** %eGFR changes over 3 years, **d** %eGFR change slope. Hazard ratios adjusted for baseline characteristics with 95% confidence intervals are indicated by *blue lines* and *gray zones*, respectively. *eGFR* estimated glomerular filtration rate, *HR* hazard ratio

**Fig. 3:**
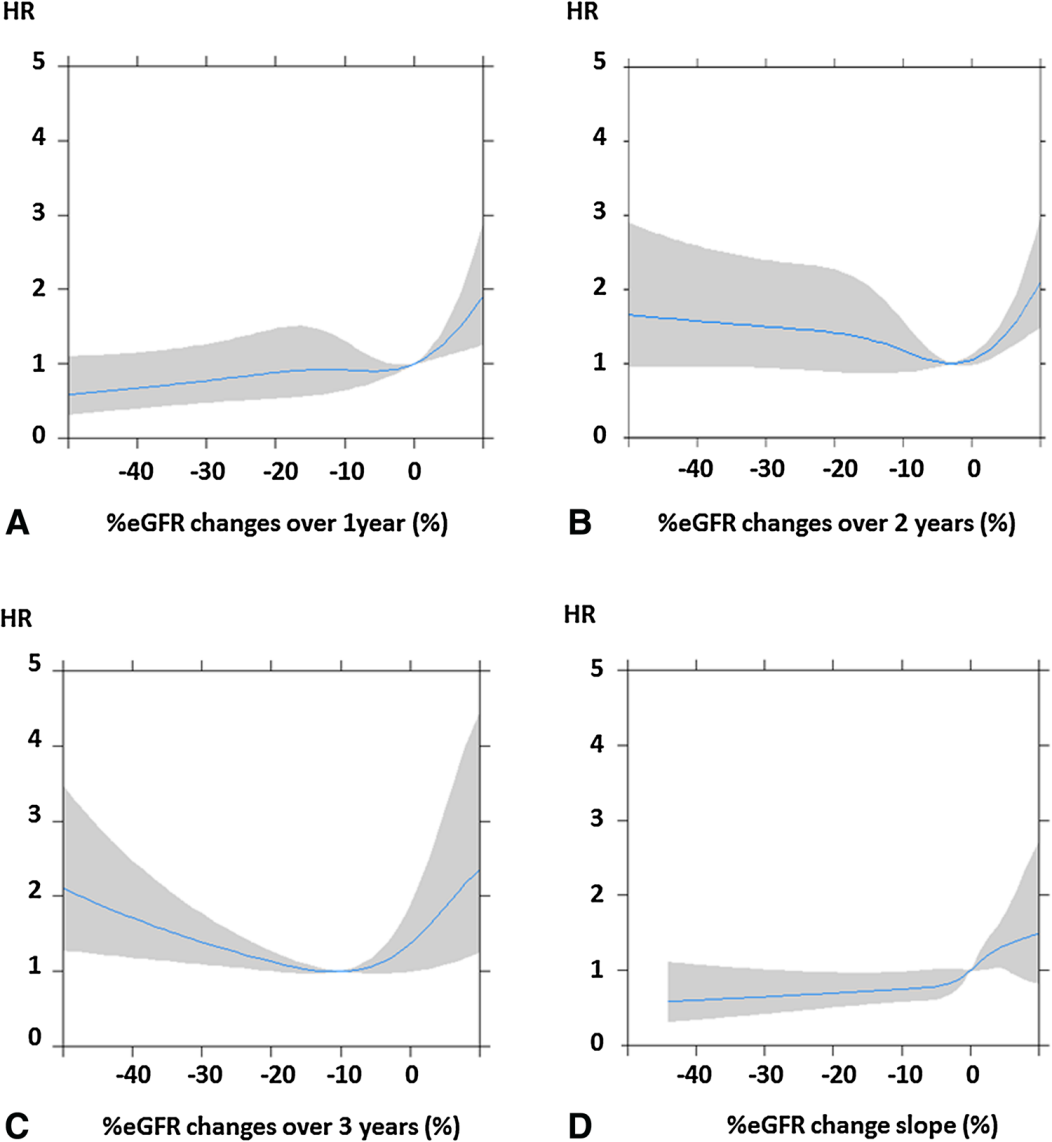
Relationships between %eGFR changes and risk of ESRD in the low-eGFR group. **a** %eGFR changes over 1 year, **b** %eGFR changes over 2 years, **c** %eGFR changes over 3 years, **d** %eGFR change slope. Hazard ratios adjusted for baseline characteristics with 95% confidence intervals are indicated by *blue lines* and *gray zones*, respectively. *eGFR* estimated glomerular filtration rate, *HR* hazard ratio

Figures 2 and 3 show the relationships between %eGFR changes and the risk of ESRD. In the high-eGFR group, the risk of ESRD tended to be high with higher rates of decreases in %eGFR changes over 2 or 3 years (Fig. 2b, c). In the low-eGFR group, the risk of ESRD also tended to be high with higher rates of decreases in %eGFR changes over 2 or 3 years (Fig. 3b, c). In the high- and low-eGFR groups, the decrease in %eGFR changes over 1 year or slope did not show a tendency to increase with the risk of ESRD (Figs. 2a, d, 3a, d). Moreover, in the low-eGFR groups, increases in %eGFR changes showed a high risk of ESRD (Fig. 3a–d).

### Accuracy of %eGFR changes for predicting ESRD

**Fig. 4:**
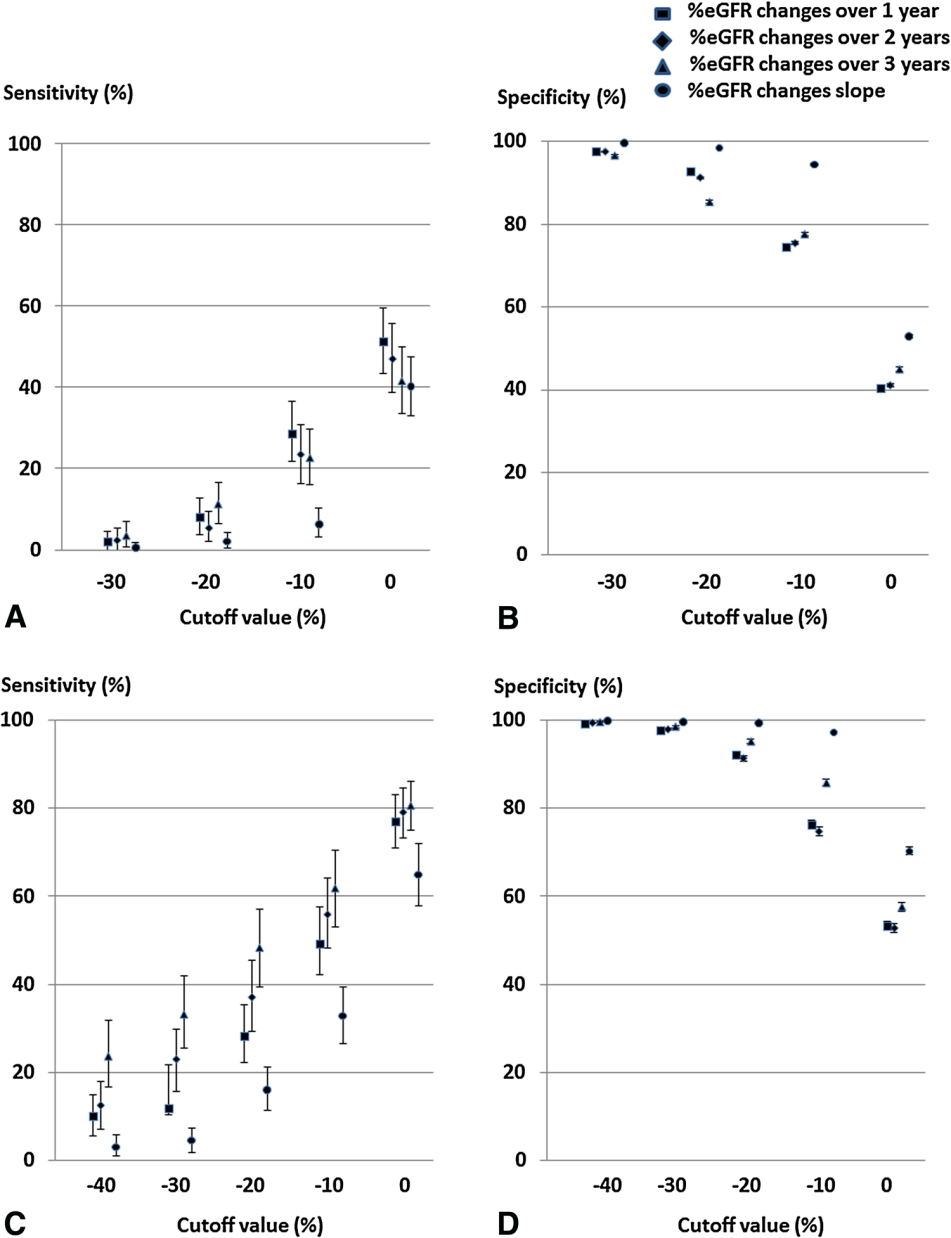
Sensitivities and specificities of ESRD based on a cutoff value of %eGFR change. **a** Sensitivities in the high-eGFR group, **b** specificities in the high-eGFR group, **c** sensitivities in the low-eGFR group, **d** specificities in the low-eGFR group. Sensitivities and specificities are shown with median and 95% confidence intervals. *ESRD* end-stage renal disease, *cutoff values* cutoff values of %eGFR change to predict ESRD

The sensitivities and specificities of %eGFR changes as cutoff values for predicting ESRD were estimated (Fig. 4). The sensitivities were low for high rates of decrease in %eGFR changes (Fig. 4a, c). Specificities were high for high rates of decrease in %eGFR changes (Fig. 4b, d). In particular, the specificities of %eGFR changes of less than −30% were more than 90% in the high- and low-eGFR groups.

**Fig. 5:**
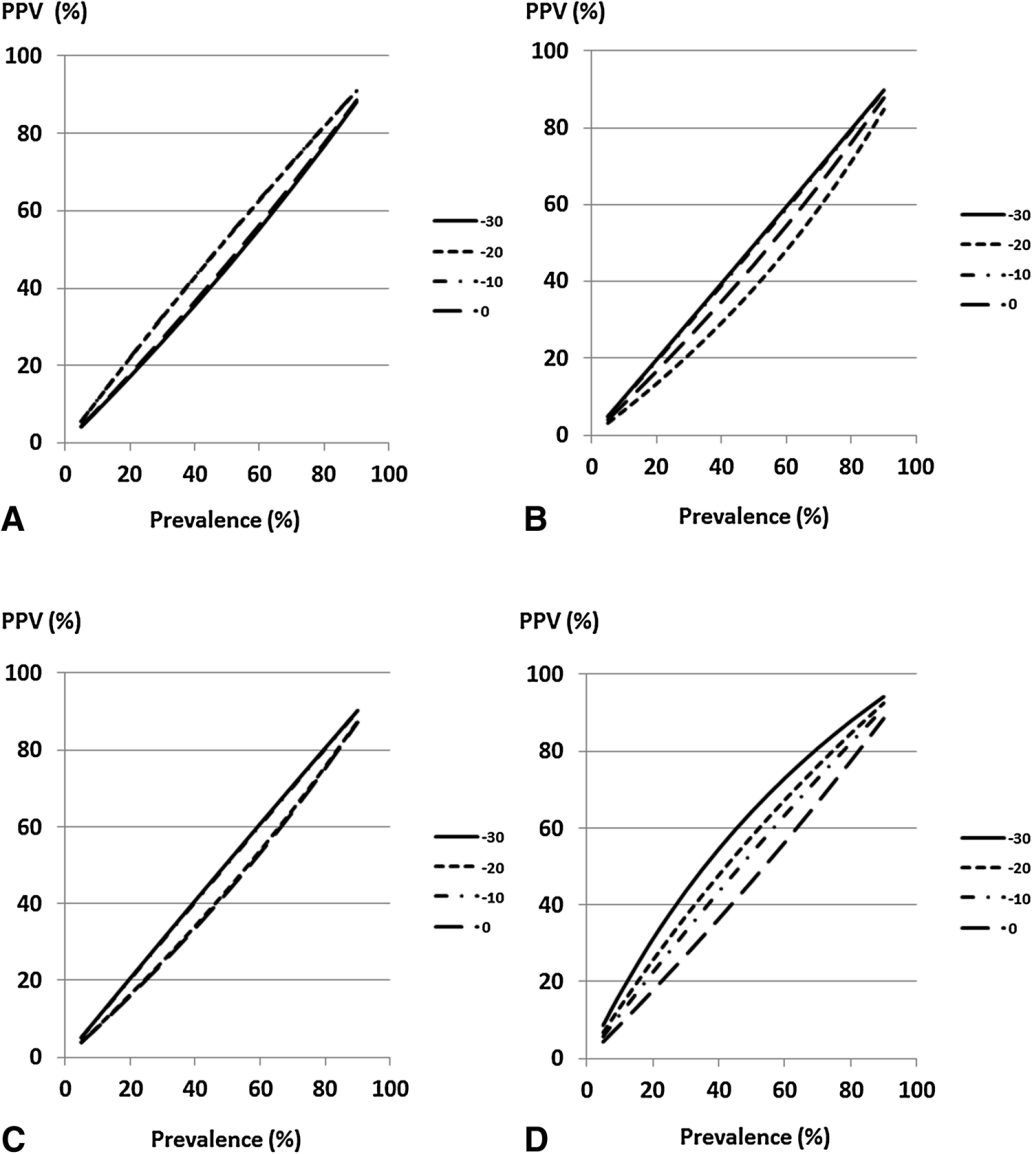
Positive predictive value of ESRD based on a cutoff value of %eGFR change in the high-eGFR group. **a** %eGFR changes over 1 year, **b** %eGFR changes over 2 years, **c** %eGFR changes over 3 years, **d** %eGFR change slope. Under conditions that the sensitivity and specificity to a cutoff value of %eGFR change were constant, PPV was obtained on the basis of change in the prevalence of subjects with a %eGFR change less than the cutoff value. *ESRD* end-stage renal disease, *eGFR* estimated glomerular filtration rate, *PPV* positive predictive value

**Fig. 6:**
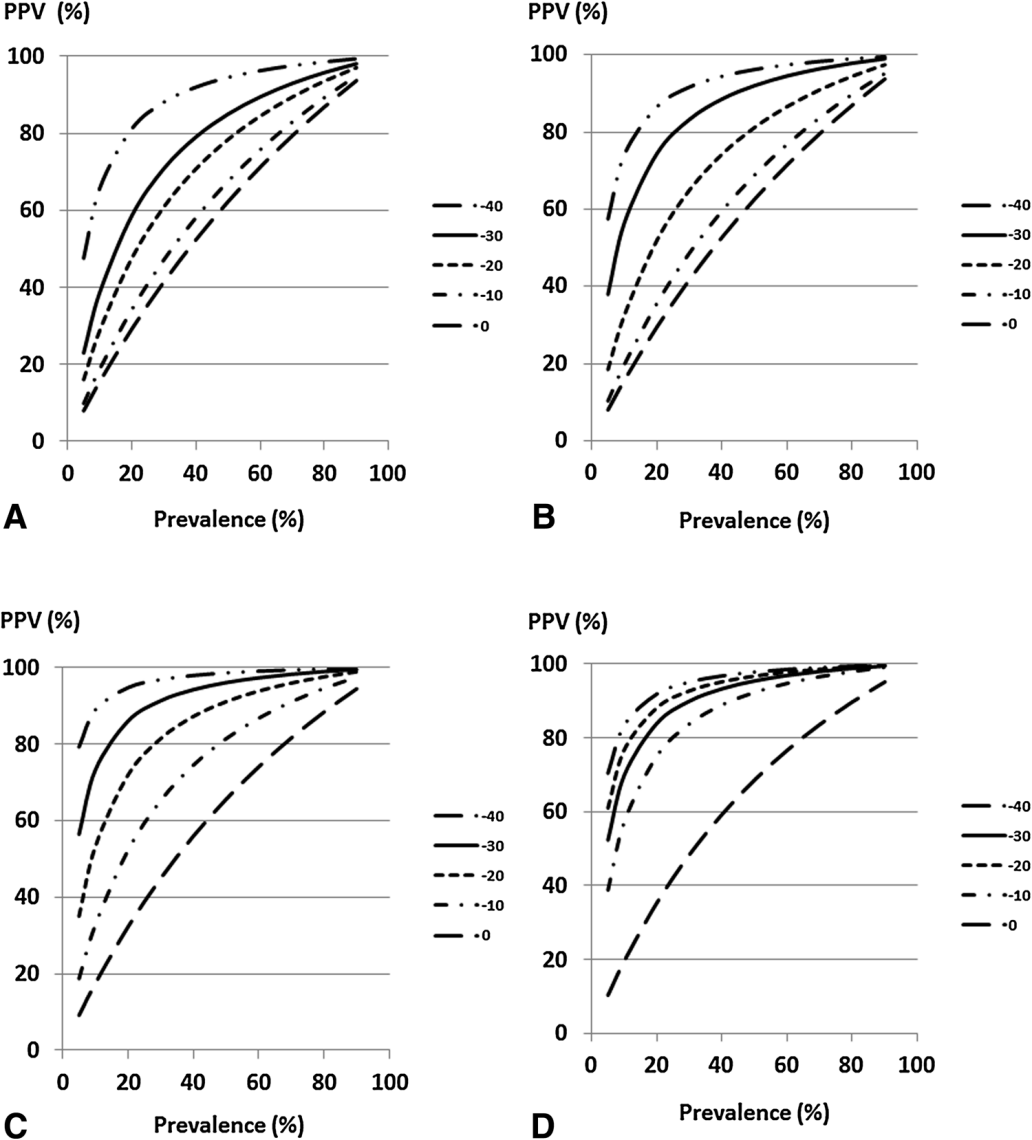
Positive predictive value of ESRD based on a cutoff value of %eGFR change in the low-eGFR group. **a** %eGFR changes over 1 year, **b** %eGFR changes over 2 years, **c** %eGFR changes over 3 years, **d** %eGFR change slope. Under conditions that the sensitivity and specificity to a cutoff value of %eGFR change were constant, PPV was obtained on the basis of change in the prevalence of subjects with a %eGFR change less than the cutoff value. *ESRD* end-stage renal disease, *eGFR* estimated glomerular filtration rate, *PPV* positive predictive value

The relationships between PPV and the prevalence of at-risk subjects at each cutoff value of %eGFR change are shown in Figs. 5 and 6. PPV increased with prevalence. In the high-eGFR group, the cutoff value of −30% showed higher PPVs than other cutoff values of %eGFR changes over 2, 3 years or slope (Fig. 5b–d). In the low-eGFR group, PPVs at cutoff values of −40 and −30% were higher than those at other cutoff values of %eGFR changes over 1–3 years (Fig. 6a–c).

## Discussion

In this study, the association between %eGFR change and ESRD was shown in the high- and low-eGFR groups. This finding was consistent with those of previous studies [2, 6, 8]. In Japan, it has been reported that 81.3% of the subjects showed GFRs of higher than 60 ml/min/1.73 m^2^, and 18.7% of the subjects showed GFRs of 60 ml/min/1.73 m^2^ or lower [14]. From the public health viewpoint, eGFR change is an important index for the prevention of CKD progression in the general population.

The criteria for the strength of a surrogate endpoint are defined by the statistical principles for clinical trials of the International Conference on Harmonization of Technical Requirements for Registration of Pharmaceuticals for Human Use (ICH) E9 as follows: (1) the biological plausibility of the relationship, (2) the demonstration of the prognostic value of the surrogate for the clinical outcome in epidemiological studies, and (3) evidence from clinical trials showing that the treatment effects on the surrogate correspond to the effects on the clinical outcome [15]. Regarding the first criterion, %eGFR change is biologically associated with ESRD because the loss of kidney function leads to ESRD; therefore, the biological plausibility of the relationship is self-evident. Thus, in this study, we mainly evaluated the second criterion.

The association between %eGFR change and ESRD has been examined in previous studies [2, 6, 8, 16]. This study showed similar relationships, and that the risk of ESRD gradually increased with the decrease in %eGFR change over 2 or 3 years in the high- and low-eGFR groups. On the other hand, in the high- and low-eGFR groups, no relationship between the decrease in %eGFR changes over 1 year and slope and a high risk of ESRD was observed. The results of previous studies and this study taken together suggest that 2 or 3 years can be the baseline period in the high- and low-eGFR groups [2, 6, 8]. Interventions, such as the use of angiotensin-converting enzyme inhibitors (ACEIs) and angiotensin II receptor blockers (ARBs), may reversibly change GFR acutely, thereby protecting kidney function [5]. Moreover, it has been reported that acute change in GFR on initiation of renin–angiotensin system blockers is weakly associated with renal outcomes [17]. Because this change in GFR observed in a short-term clinical trial may be temporary and acute, a long-term observation period is required to accurately evaluate the improvement of GFR. Therefore, the baseline period over 2 or 3 years would be better for the unit of %eGFR change than the baseline period over 1 year. The US National Kidney Foundation and the US Food and Drug Administration reported that “a confirmed decline in estimated GFR of 30% over 2–3 years may be an acceptable surrogate end point” [7]. Because there is a possibility that the optimal baseline period may differ on the basis of the characteristics of the subjects, more studies of various populations are needed to determine surrogate endpoints.

For %eGFR change to be used as a surrogate endpoint of ESRD, its cutoff value should be determined. In this study, the number of the subjects with %eGFR changes of less than −40% was small. The distribution of the risk of ESRD showed that in the high- and low-eGFR groups, the risk of ESRD was higher in %eGFR changes of less than −30% over 2 or 3 years than in other ranges of %eGFR changes. These findings were in accordance with those of previous studies [2, 6, 8]. These lines of evidence indicate that persons with %eGFR changes of less than −30% over 2 or 3 years have a high risk of ESRD.

To predict accurately a true endpoint, it is better that the specificity of a surrogate endpoint should be high. In this study, in the high- and low-eGFR groups, the specificities of %eGFR changes of less than −30% were more than 90%. Moreover, the relationship between a surrogate endpoint and a true endpoint is related to the proportion of persons with the surrogate endpoint who will have the true endpoint in the future. That is, PPV is based on a similar concept to a surrogate endpoint, and a good surrogate endpoint has a high PPV. In this study, the PPVs based on %eGFR changes of −30% over 2 or 3 years were higher than those based on other %eGFR changes in the high- and low-eGFR groups. These findings suggest that %eGFR changes of −30% over 2 or 3 years had the highest substitutability for ESRD. Therefore, −30% can be a candidate cutoff value for ESRD in the high- and low-eGFR groups. The doubling serum creatinine level is equivalent to −53% decline in eGFR, as determined on the basis of the JSN’s eGFR equation. Because the subjects with %eGFR changes of less than −53% were very rare in our study, the observation of doubling serum creatinine level may be difficult in a community-based study.

This study lacked information on the participants’ death, and the persons who died during the course of this study were not included in the analysis. CKD in the majority of affected patients does not progress to ESRD because such patients die before the onset of ESRD [18–21]. Because we treated ESRD only as the outcome, we were unable to control for competitive risk bias. However, the subjects of this study were persons who underwent annual community-based health examinations and were healthier than usual CKD patients. The number of subjects with a high risk of death and with comorbidities, such as cardiovascular disease and diabetes mellitus, and the number of elderly CKD patients was small: therefore, the competitive risk bias might be small. Although the %eGFR changes in this study might have been different from those in CKD patients with various comorbidities, our study showed similar relationships to previous studies [2, 6, 8, 16]. Regardless of the bias, our findings suggest that the relationships between the risk of ESRD and %eGFR change are robust. Moreover, these findings showed the existence of high risk persons in the general Japanese population who needed care of their kidney function.

In this study, in the low-eGFR group, a high risk of ESRD was associated with increases in %eGFR changes. This suggests that some of the subjects showing increases in %eGFR changes may have a high risk of ESRD. eGFR is estimated from serum creatinine level. Serum creatinine level is associated with skeletal muscle mass, and the weight-loss-associated decrease in skeletal muscle mass increases eGFR [22–24]. Moreover, a cohort study of a nondiabetic population showed that prediabetes is associated with glomerular hyperfiltration [25]. Considering these lines of evidence, some of the subjects showing increases in %eGFR changes are not always healthy but may have some comorbidities that lead to a decrease in muscle mass or diabetes mellitus.

This study has several limitations. First, because of the observational nature of this study, the results may be biased owing to unmeasured confounders. Second, we did not include the patients with missing data in this study. This might have caused selection bias. Third, because the number of ESRD patients was small, we were unable to investigate stratifications of baseline characteristics such as gender, old age, and urinary protein level. Fourth, comorbidities such as malnutrition and diabetes mellitus and medications such as ACEIs and ARBs were not investigated in this study. The effects of these factors on eGFR were not evaluated. Fifth, the number of subjects with %eGFR change of 0 to less than 10% over 1–3 years was smaller than those of the subjects with other %eGFR changes over 1–3 years in the high- and low-eGFR groups. There was a possibility that the rounding error of serum creatinine level might have caused the unbalanced distribution of %eGFR changes over 1–3 years. Sixth, the prediction capability of %eGFR change is not necessary applicable in the clinical practice as long as 15 years in the future, because interventions to the many risk factors of the CKD progression may be developed currently.

## Conclusions

This study showed that the relationship between %eGFR change and the risk of ESRD. Moreover, %eGFR changes of less than −30% over 2 or 3 years may be candidate surrogate endpoints for ESRD in the general Japanese population.
